# Evaluation of a rapid one-step PSA test for primary prostate cancer screening

**DOI:** 10.1186/s12894-021-00903-7

**Published:** 2021-09-27

**Authors:** Shingo Ashida, Ichiro Yamasaki, Chiaki Kawada, Hideo Fukuhara, Satoshi Fukata, Kenji Tamura, Takashi Karashima, Keiji Inoue, Taro Shuin

**Affiliations:** 1 Department of Urology, Kochi Medical School, Nankoku, Kochi 783-8505 Japan; 2 Department of Urology, Kubokawa Hospital, Takaoka-gun, Kochi 786-0002 Japan; 3 Kochi Medical School Hospital, Nankoku, Kochi 783-8505 Japan

**Keywords:** Prostate cancer, Screening, Prostate-specific antigen, Rapid test

## Abstract

**Background:**

To enhance the convenience and reduce the cost of prostate cancer (PC) screening, a one-step prostate-specific antigen (PSA) test was evaluated in a large population. The PSA SPOT test kit enables rapid detection of human PSA in serum or plasma at or above a cutoff level of 4 ng/mL to aid in the diagnosis of PC.

**Methods:**

PC screening using the PSA SPOT test was offered to male participants in educational public lectures that we conducted in various cities. Test results were reported to participants at the end of the lectures. Blood samples from 1429 men were evaluated. Two independent observers interpreted the tests at 15 and 30 min. The remaining serum samples were subsequently tested using a conventional quantitative assay.

**Results:**

The sensitivity, specificity, positive predictive value, negative predictive value, and accuracy of the test were 79.9, 93.0, 65.4, 96.6, and 91.2%, respectively. The sensitivity and specificity of the test changed with variations in the reading time. Quantitative assessment of the intensity of the band was correlated with the PSA value.

**Conclusions:**

PSA testing using this kit can be easily performed. The low cost and speed of the test make it a useful and convenient tool for primary PC screening.

## Background

Prostate cancer (PC) is one of the most common malignancies and causes of cancer death among men worldwide [1], with an estimated 78,500 new cases in 2019 in Japan. The widespread use of the prostate-specific antigen (PSA) test is thought to be responsible for the rapid increase in PC diagnoses between 1988 and 1992 in the United States [2, 3].

Both the high prevalence of PC and availability of PSA tests capable of detecting PC at an early stage are important criteria required to support mass screening. The significant increase in the diagnosis of organ-confined PC—which means a reduction in metastatic disease—justifies PC screening [4–6]. Moreover, recent randomized clinical trials (RCTs) demonstrated a 21 to 44% decline in PC mortality rates due to PSA screening [7, 8], which could represent one of the most persuasive arguments in support of PC screening.

However, PC screening is still controversial, as the potential benefits and harms continue to be debated among health professionals. Major controversies regarding PC screening center on the possibilities of over-diagnosis and over-treatment. Some patients might suffer from complications associated with the treatment of clinically insignificant PCs that would probably never lead to death. Another issue involves the economics of PC screening, including the costs associated with detection, treatment, and treatment-related complications. Optenberg and Thompson estimated that the cost of screening could be as high as $25 billion annually if all men 50–70 years of age in the United States participated in a screening program [9].

In this study, we evaluated a rapid, one-step, qualitative PSA test, called the PSA SPOT test, as a possible way to enhance the convenience and reduce the cost of PC screening in a large population.

## Methods

### PSA SPOT test

**Fig. 1:**
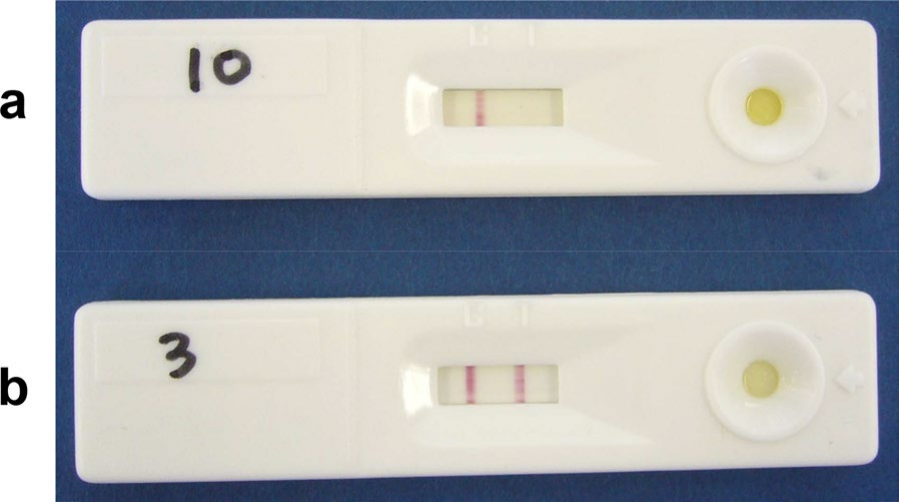
PSA SPOT test. **a** No band appears in the test zone (negative). **b** A red-colored band appears in the test zone (positive)

The PSA SPOT test was performed according to the instructions provided by Prof. Rajvir Dahiya, PhD, Department of Urology, Veterans Affairs Medical Center and University of California, San Francisco, California. The format of the PSA SPOT test is a double-antibody sandwich. Antibodies specific to PSA are conjugated to colloidal gold and incorporated into a strip pad, and these antibodies capture any PSA after the sample is added to the test strip. The antigen-antibody-gold complexes migrate along the nitrocellulose membrane via capillary action and are then captured by marker-specific antibodies immobilized on the membrane. A red-colored band will appear in the test zone (T) if PSA protein is present in the specimen (Fig. 1). Antibody-gold complexes are captured in the control zone (C), where goat anti-mouse IgG is immobilized. To serve as an internal process control, a red-colored control band was designed to appear as an indication that the test was performed properly and should always be seen after the test is completed. The absence of a red control band in the control region is an indication of an invalid result.

The PSA SPOT test kit is designed for the rapid detection of human PSA in serum or plasma at or above a cutoff level of 4 ng/mL. In the present study, two independent observers interpreted the test results at 15 and 30 min. The remaining serum samples were subsequently tested using a conventional quantitative assay (Access Hybritech PSA, Beckman Coulter, Inc.). The tests were interpreted as negative if no band appeared in the test region; if a band developed, the test was judged as either weakly positive (+) or strongly positive (++) according to the intensity of the color reaction.

### Validation of the PSA SPOT test

To validate the PSA SPOT test, serum samples obtained from 53 consecutive patients suspected of having or followed up for PC were tested using the PSA SPOT test and quantitative PSA test (Access Hybritech PSA). The sensitivity and specificity of the PSA SPOT test were determined using the cut-off value of 4.0 ng/mL.

### PC screening using the PSA SPOT test

PC screening using the PSA SPOT test kit was offered to male participants in educational public lectures that we conducted in various cities from June 2005 to July 2016. Blood samples from 1429 men (mean age, 70.8 years; range, 30–93 years) were evaluated. The blood samples were obtained before the lectures, serum was separated from blood by centrifugation, and the PSA SPOT test was immediately performed. The results of the tests were reported to the participants at the end of the lectures. We then enclosed and passed referral letters if the results were judged as positive.

This study was approved by the ethical review board of Kochi Medical School (ethical approval no. 16–12). Written informed consent was obtained regarding PC screening using the PSA SPOT test. All methods were carried out in accordance with relevant guidelines and regulations.

### Statistical analysis

All statistical analyses were performed using JMP® software (SAS Institute Inc., Cary, NC, USA). A *P* value < 0.05 was considered to indicate a statistically significant difference. The sensitivity, specificity, positive predictive value (PPV), negative predictive value (NPV), and accuracy of the PSA SPOT test were calculated. A box-and-whisker plot was used to show the correlation between the PSA quantitative value and intensity of the color reaction band on the PSA SPOT test. Pearson’s correlation test was used to correlate the two variables described above.

## Results

In the validation series using serum samples from 54 patients suspected of having or followed up for PC, the sensitivity and specificity of the PSA SPOT test were 9.1 and 96.8%, respectively, at a reading time of 15 min and 90.9 and 93.5%, respectively, at 30 min.

Of 1429 participants, 1223 (85.6%) had a PSA value of less than 4 ng/mL (median PSA, 1.270 ng/mL; range, 0.001–3.972 ng/mL), and 206 (14.4%) presented a PSA value higher than 4 ng/mL (median PSA, 6.304 ng/mL; range, 4.018–237.518 ng/mL). A total of 164 (11.5%) participants had a PSA value between 4 and 10 ng/mL.

The results of the test were affected by variations in the reading time. The sensitivity was very low at a reading time of 15 min (41.7%) but increased at a reading time of 30 min (79.9%), whereas the specificity of the test was similar (98.7% at 15 min, 93.0% at 30 min). Thus, we chose 30 min as the optimal reading time for further analyses.

The sensitivity, specificity, PPV, NPV, and accuracy of the test were 79.9, 93.0, 65.4, 96.6, and 91.2%, respectively. Among the 1151 participants with a PSA value < 4 ng/mL, 1071 were correctly interpreted as negative using the PSA SPOT test, whereas 80 were interpreted as positive, resulting in a test specificity of 93.0%. Of the 80 false-positive results, 39 (48.8%) were in the PSA range of 3–4 ng/mL (Table 1). The specificity of the test in the PSA range of 3–4 ng/mL was 63.9% (69/108).

**Table 1:** Correlation of PSA SPOT test interpretations with PSA quantitative values

PSA (ng/mL)	n*	PSA SPOT test			Accuracy (%)
		−	+	++	
< 3	1043	1002	32	9	96.1
3–4	108	69	23	16	63.9
4–5	52	23	12	17	55.8
5–10	97	14	22	61	85.6
10–20	23	0	1	22	100.0
> 20	17	1	0	16	94.1

^*^Some samples not included due to invalid results

**Fig. 2:**
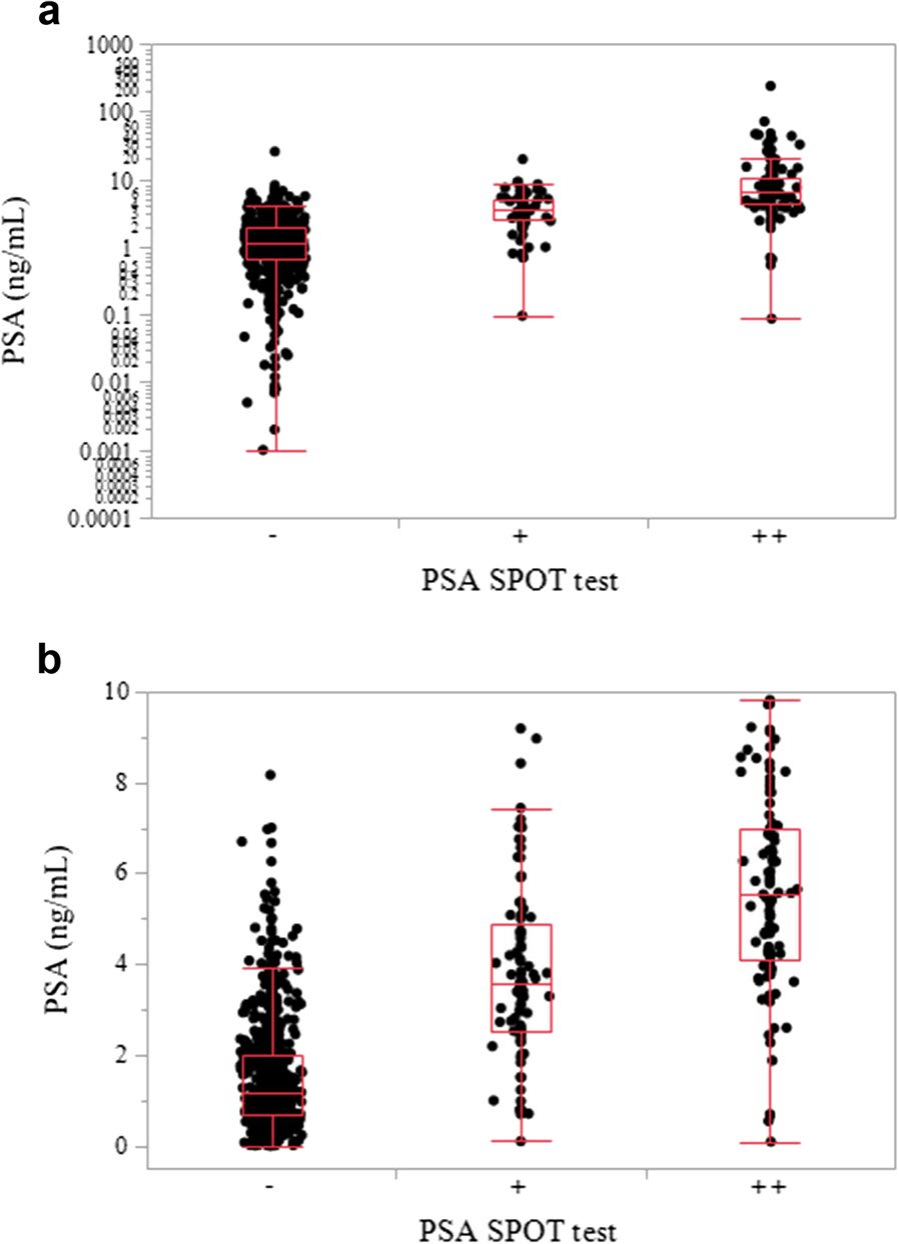
Box-and-whisker plots. PSA quantitative values were plotted according to the intensity of the red bands in PSA SPOT tests among all samples (**a**) and samples in which the PSA value was between 0 and 10 ng/mL (**b**). The horizontal line within each box represents the median, the ends of the box represent the 25th and 75th percentiles, the whiskers extend from the ends of the box to the minimum and maximum, and the individual dots beyond the whiskers represent outliers

Of 151 true positive results, 112 exhibited a PSA value between 4 and 10 ng/mL; 34/112 (30.4%) were interpreted as weakly positive by the PSA SPOT test, whereas 78/112 (69.6%) were judged as strongly positive (Table 1). A total of 40 participants presented a PSA value > 10 ng/mL; 1/40 (2.5%) was judged as weakly positive, whereas 38/40 (95.0%) were judged as strongly positive. As shown in Fig. 2, the intensity of the color reaction was correlated with the PSA quantitative value (Pearson’s correlation test: *r* = 0.39, *P* < 0.0001).

## Discussion

Since Catalona et al. first demonstrated in 1991 that determination of PSA could be used as a first-line screening test for PC in men without suspicious digital rectal examination findings [10], PSA testing has been widely applied. This has resulted in a spike in PC incidence rates, as previously undetectable cases of PC were unmasked.

Although mass screening for PC remains one of the most controversial issues in oncology, two large, high-quality RCTs were carried out to evaluate PSA screening. The Swedish Göteborg trial demonstrated a 44% lower PC mortality rate in the screening arm among men aged 55 to 69 years over approximately 14 years [7]. The European Randomized Study of Screening for Prostate Cancer (ERSPC) reported a 21% reduction in PC mortality in the screening arm during 13 years of follow-up [8].

The controversy over PSA screening includes the possibilities of detecting insignificant PCs that would never lead to death (over-diagnosis) and then treating these PCs (over-treatment). Other issues include cost and convenience, which are possible reasons why the rate of PSA screening is low in Japan. To overcome these issues, we conducted educational public lectures in Japanese cities and offered a one-step PSA test for free.

Several reports have described rapid, one-step, qualitative PSA tests, as summarized in Table 2 [11–16]. However, the sample sizes of these studies have been limited, with the exception of the study of Berg et al., which reported a specificity (81%) that appears to be unacceptable. Therefore, we evaluated a one-step PSA test in a large population of Japanese males to potentially enhance the convenience and reduce the cost of PC screening. The one-step PSA test we describe is very easy to administer and can be performed without costly additional equipment, although centrifuge is needed to separate serum from blood. The low cost and speed of the test make it useful and convenient as a tool for primary PC screening, even in general practitioner or urologist office settings. The economic drawbacks to PSA mass screening could be overcome using this one-step PSA test. In general, a rapid test can be priced much lower because it utilizes a simple method [16]. Therefore, the use of this test could spare a significant number of costly quantitative PSA determinations in screening populations due to the high percentage of negative PSA results in PC mass screening, although we could not show expected cost savings because the PSA SPOT test kit is not commercially available and not priced.

**Table 2:** One-step PSA tests reported in the literature

Author	n	PSA test	Sensitivity (%)	Specificity (%)
Madersbacher et al.	238	Oncoscreen	93	93
Jung et al.	99	Chembio	67	87
Jung et al.	99	Medpro	87	88
Jung et al.	99	Seratec	80	97
Jung et al.	99	Syntron	93	93
Dok An et al.	147	One Step PSA	100	90
Berg et al.	2322	Uralen	91	81
Miano et al.	188	PSA RapidScreen	97.6	90.4
Shigeno et al.	614	PSA Rapid Test	89.5	94.2

Although the overall specificity was 93.0% in our study, it was lower in the PSA range 3–4 ng/mL, within which false-positive results were quite common (36.1%). The results of the PSA SPOT test were comparable to those of other one-step PSA tests described in the literature (Table 2). The primary limitations of this test appear to be poor accuracy in the PSA range 3–4 ng/mL. As such, greater precision is needed to minimize the number of false-positive results. The secondary limitations might be poor accuracy in the PSA range 4–5 ng/mL. The overall sensitivity will increase to 89.1% if the PSA cut-off value of 5.0 ng/mL is used. Nonetheless, the one-step PSA SPOT test could be useful in the general practitioner or urologist office setting as well as in mass screening. If the test could be modified to allow administration at home, PSA testing might be reassessed as a means of mass screening for PC.

## Conclusions

The PSA SPOT test is a simple, feasible, and reproducible tool for PC screening. The lower cost, ease of handling, and rapid procedure could make this test useful in the general practitioner or urologist office setting as well as for mass primary PC screening.

## Data Availability

The datasets used and/or analyzed during the current study are available from the corresponding author on reasonable request.

## Abbreviations

PC: Prostate cancer
PSA: Prostate-specific antigen
RCTs: Randomized clinical trials
PPV: Positive predictive value
NPV: Negative predictive value
ERSPC: European Randomized Study of Screening for Prostate Cancer

## References

[1] Kimura T, Egawa S (2018). Epidemiology of prostate cancer in Asian countries. Int J Urol Off J Jpn Urol Assoc.

[2] Potosky AL, Miller BA, Albertsen PC, Kramer BS (1995). The role of increasing detection in the rising incidence of prostate cancer. JAMA.

[3] Siegel RL, Miller KD, Jemal A (2019). Cancer statistics, 2019. CA Cancer J Clin.

[4] Catalona WJ, Richie JP, Ahmann FR, Hudson MA, Scardino PT, Flanigan RC, DeKernion JB, Ratliff TL, Kavoussi LR, Dalkin BL (1994). Comparison of digital rectal examination and serum prostate specific antigen in the early detection of prostate cancer: results of a multicenter clinical trial of 6630 men. J Urol.

[5] Crawford ED (1997). Prostate cancer awareness week: September 22 to 28, 1997. CA Cancer J Clin.

[6] Stephenson RA, Stanford JL (1997). Population-based prostate cancer trends in the United States: patterns of change in the era of prostate-specific antigen. World J Urol.

[7] Hugosson J, Carlsson S, Aus G, Bergdahl S, Khatami A, Lodding P, Pihl CG, Stranne J, Holmberg E, Lilja H (2010). Mortality results from the Göteborg randomised population-based prostate-cancer screening trial. Lancet Oncol.

[8] Schröder FH, Hugosson J, Roobol MJ, Tammela TL, Zappa M, Nelen V, Kwiatkowski M, Lujan M, Määttänen L, Lilja H (2014). Screening and prostate cancer mortality: results of the European Randomised Study of Screening for Prostate Cancer (ERSPC) at 13 years of follow-up. Lancet (Lond, Engl).

[9] Optenberg SA, Thompson IM (1990). Economics of screening for carcinoma of the prostate. Urol Clin N Am.

[10] Catalona WJ, Smith DS, Ratliff TL, Dodds KM, Coplen DE, Yuan JJ, Petros JA, Andriole GL (1991). Measurement of prostate-specific antigen in serum as a screening test for prostate cancer. N Engl J Med.

[11] Berg W, Linder C, Eschholz G, Schubert J (2001). Pilot study of the practical relevance of a one-step test for prostate-specific antigen in capillary blood to improve the acceptance rate in the early detection program of prostate carcinoma. Int Urol Nephrol.

[12] Dok An C, Yoshiki T, Lee G, Okada Y (2001). Evaluation of a rapid qualitative prostate specific antigen assay, the One Step PSA(TM) test. Cancer Lett.

[13] Jung K, Zachow J, Lein M, Brux B, Sinha P, Lenk S, Schnorr D, Loening SA (1999). Rapid detection of elevated prostate-specific antigen levels in blood: performance of various membrane strip tests compared. Urology.

[14] Madersbacher S, Mian C, Maier U, Simak R (1996). Validation of a 10-minute dipstick test for serum prostate-specific antigen. Eur Urol.

[15] Miano R, Mele GO, Germani S, Bove P, Sansalone S, Pugliese PF, Micali F (2005). Evaluation of a new, rapid, qualitative, one-step PSA Test for prostate cancer screening: the PSA RapidScreen test. Prostate Cancer Prostatic Dis.

[16] Shigeno K, Arichi N, Yoneda T, Kishi H, Shiina H, Igawa M (2006). Usefulness of an immunochromatographical assay, PSA Rapid Test as a primary screening test for prostate cancer. Int Urol Nephrol.

